# Good practice recommendations to better coordinate the management of oncological pain: a Delphi survey

**DOI:** 10.1038/s41598-022-26753-3

**Published:** 2022-12-28

**Authors:** Yolanda Escobar, César Margarit, Concepción Pérez-Hernández, Teresa Quintanar, Juan A. Virizuela

**Affiliations:** 1 Medical Oncology Service, Spanish Medical Oncology Society, University General Hospital Gregorio Marañón, Madrid, Spain; 2 Pain Unit, Spanish Pain Society, University General Hospital of Alicante, Alicante, Spain; 3 Pain Unit, Spanish Pain Society, University Hospital de la Princesa, Madrid, Spain; 4 Medical Oncology Service, Spanish Medical Oncology Society, University General Hospital of Elche, Alicante, Spain; 5 Medical Oncology Service, Spanish Medical Oncology Society, University Hospital Virgen Macarena, Sevilla, Spain

**Keywords:** Cancer, Health care

## Abstract

Treatment of oncological pain is complex and requires a multidisciplinary management approach between oncology services and pain units. Although significant improvements have been achieved in the treatment and overall survival of cancer patients, the management of oncological pain has not followed the same directions. Many patients are not referred to pain units even though they could benefit from it. The purpose of this Delphi survey was to map the current situation in the management of cancer pain, identify barriers and propose recommendations to improve its management by emphasizing the importance of collaboration and coordination between oncology services and pain units. A survey among members with recognized experience in the management of oncology patients and oncological pain was held based on the Delphi method principles. The experts were asked to vote preselected statements on cancer pain management in two rounds and conclusions and recommendations were formulated based on the consensus reached for each statement. Barriers and areas for improvement were identified: need of multidisciplinary management approach, effective communication between oncology services and pain units, timely referral of cancer patients to pain units, training of health care professionals dealing with cancer aspects and identification of those patients that could benefit from a multidisciplinary management of their oncological disease. The experts issued recommendations targeting the identified barriers and areas for improvement by defining the service requirements of hospital and units treating cancer pain patients, establishing referral pathways necessities and adopted measures to improve the care of cancer patients.

## Introduction

Cancer is a devastating diagnosis equally for patients and their families^1^. Cancer diagnosis is almost always synonymous with pain, which affects life quality of patients, their family and caregivers^2^. Pain can be present even in early disease stages and its frequency and intensity increase progressively affecting almost 90% of patients during its late stages^3,4^. Effective pain treatment is essential in the overall management of cancer patients and the importance of controlling cancer pain effectively cannot be overemphasized^5^. Aggressive and meticulous pain control prolongs cancer patients’ survival^6^.

Many patients still suffer from significant amount of pain, as analgesic treatments might be inadequate^7^. Interestingly, this lack of adequate pain control either due to cancer or its complications or its association with surgical procedures did not improve significantly over the years^8^. Ineffective cancer pain control is a multifaceted issue and its adequate evaluation depends on a collaborative interaction between patients and healthcare professionals.

Pain is subjective and, often, patients do not emphasize it adequately believing that their doctors will divert their attention to the symptom rather than focusing on main disease treatments^9^. Also health care professionals have often limited knowledge and skills when using opioids, a cornerstone in the pharmacotherapy of cancer pain, which together with local opioid accessibility problems, contributes importantly to the under management of the symptoms.

Recent surveys have identified a need for education regarding pain practices amongst the various professionals involved in cancer pain treatments^10^. A review published by Kwon regarding barriers in cancer pain management highlighted important topics like opioid rotation and appropriate breakthrough pain handling as suitable educational targets to improve outcomes^4^.

Cancer pain management can be quite complex because pain is often accompanied by emotional problems and a variety of other symptoms. Therefore, a multidisciplinary approach is required to assess and manage patients suffering from it^11^. An extensive collaboration and coordination amongst providers and optimal use of available therapeutic options are necessary, together with accurate technical information exchange and frequent communication flow amongst not only the health care professionals involved in the management of cancer pain but the patients as well^12^.

Our aim was to outline current trends in oncological pain management, recognize barriers and recommend measures to improve the situation by:

1. Identifying the necessary resources that an institution or clinical service should be equipped with to effectively manage cancer pain patients.
2. Proposing referral pathways and coordination/communication processes between the country's pain units and oncology services for an effective treatment and follow up of cancer pain patients.
3. Introducing training opportunities for the health care professionals handling cancer pain cases to improve therapeutic outcomes and consequently life quality of affected patients.

## Methods

### Expert panel selection

The Spanish Medical Oncology Society (SEOM) and the Spanish Society of Pain (SED) promoted the initiative and designated 3 representatives from each one to appoint a lead scientific committee. This committee was composed of experts with recognized experience in clinical oncology and oncological pain management.

The scientific committee generated statements/items, focusing on patients, healthcare providers and healthcare system perspectives, addressing current issues and identifying barriers in the various management stages of cancer pain. These statements were sent for assessment to an expert panel consisting of 35 members, who were selected by the scientific committee and all were SEOM and SED members. They were chosen taking into account their recognized experience, professional prestige and publications in their reference field respectively.

### Study design

A Delphi method was used, whose aim is to transform individual opinions into an expert group consensus^13^. After an exhaustive literature review and discussion, the scientific committee generated 79 debatable items distributed in three content blocks: general considerations on the cancer pain management, referral criteria to a pain unit, and barriers and opportunities for the improvement for cancer pain management improvement.

The items were sent to the panelists for an online evaluation and validation by voting in two rounds. Panelists assessed the items using a single 9-point Likert-type ordinal scale, according to the model developed by the UCLA-RAND Corporation for the comparative assessment and prioritization between different health options (minimum 1 = full disagreement; and maximum 9 = full agreement)^13^.

To analyze the group’s opinion and the consensus degree reached on each question, the median and the interquartile range of the scores obtained for each item were used.

Those items that did not reach consensus (in favor or against) in the first round were reformulated and included again in the second round questionnaire. In this second round the panelists received the first survey results so that they could contrast their personal opinions with those of their fellow panelists and, if necessary, reconsider their initial opinion.

Results are shown in tables as median and IQR of the panelists’ responses and degree of agreement. Taking into account the consensus statements, the scientific committee developed a table of conclusions and recommendations on the management of cancer pain patients.

## Results

The questionnaire consisted of 79 items addressing the current coordination status between pain units and medical oncology services in Spain regarding cancer pain patients treatments, and proposed measures to improve patient flow and collaboration between pain units and medical oncology services for better therapeutic outcomes and life quality of the affected patients (Tables 1, 2, 3).

**Table 1:** Block I results.

	Median (IQR)*	Degree of agreement	Result
1. There are many cancer pain patients not referred to pain units even though they could benefit from it	8 (6–9)	75.0%	Agreement in 1st round
2. There is an excess of cancer patients’ referrals to pain units	2 (1–2)	90.6%	Disagreement in 1st round
**All hospitals that manage cancer patients must have**			
3. At least one type I pain unit (single specialist unit)	7 (3–9)	56.7%	No consensus
4. At least one type II pain unit (unit of several unidisciplinary specialists for the treatment of pain)	8 (7–9)	76.7%	Agreement in 2nd round
5. At least one type III pain unit (multidisciplinary unit for the treatment of pain)	7 (5–8)	63.3%	No consensus
6. A type IV pain unit (multidisciplinary unit with research)	6.5 (4–8)	50.0%	No consensus
7. A pain unit of any kind, or failing that, a referral unit in another hospital center, where patients for pain treatment can be referred to	9 (8–9)	87.5%	Agreement in 1st round
8. A hospital oncological pain commission, where members of the oncology services and pain units participate to	8 (7–9)	81.3%	Agreement in 1st round
**The medical oncology service and the pain unit of a hospital center must have**			
9. Shared clinical sessions where cases of cancer pain are discussed	8 (7–9)	81.3%	Agreement in 1st round
10. Shared protocols for cancer pain management	9 (8–9)	100.0%	Agreement in 1st round
11. Consensual protocols for referring the patient to the pain unit	9 (8–9)	100.0%	Agreement in 1st round
12. The possibility of quick telephone contact to consult cancer pain cases	9 (8–9)	93.8%	Agreement in 1st round
13. The possibility of consulting cases of cancer pain through telecommunications technology	8 (7–9)	84.4%	Agreement in 1st round
**In a hospital where there is a medical oncology and a pain unit, it should be possible that**			
14. Cancer pain cases are discussed in the tumor committee	9 (6–9)	73.3%	Agreement in 2nd round
15. The doctors of the medical oncology service are familiar with the portfolio of services of their referral pain unit	9 (8–9)	100.0%	Agreement in 1st round
16. The medical oncology services organize courses or training and updated sessions for the pain unit’s doctors of their center regarding fundamental principles of cancer and its treatments	8 (8–9)	93.8%	Agreement in 1st round
17. Pain units organize courses or training sessions for the medical oncology service doctors of their center regarding treatment of cancer pain	9 (8–9)	93.8%	Agreement in 1st round
**Resident physicians of a medical oncology service should receive formal training in**			
18. Pharmacological treatment of cancer pain	9 (9–9)	100.0%	Agreement in 1st round
19. Invasive anesthetic techniques for the treatment of pain, its basic principles and most common techniques such as: blocks, radiofrequency, neurostimulation and spinal or epidural infusion	8 (7–9)	84.4%	Agreement in 1st round
20. Psychological techniques	8 (7–9)	87.5%	Agreement in 1st round
21. Physiotherapy techniques	6 (4–8)	50.0%	No consensus
22. Administration of analgesic drugs using patient controlled analgesia (PCA)	8 (7–9)	87.5%	Agreement in 1st round
23. Use of the chronic pain evaluation questionnaires	8.8 (8–9)	87.5%	Agreement in 1st round
**Anesthesiology residents and pain physicians should receive regulated training in**			
24. Cancer and its treatments	8 (8–9)	75.0%	Agreement in 1st round
25. Communication with the cancer patients and their families	9 (7–9)	90.6%	Agreement in 1st round
26. The services portfolio of pain units should be public and easily accessible by any hospital specialty	9 (8–9)	96.9%	Agreement in 1st round

Cancer pain management. General considerations.

*IQR interquartile range.

**Table 2:** Block II results.

	Median (IQR)*	Degree of agreement	Results
**In your opinion**			
27. There are clear criteria for referring cancer pain patients to pain units	4 (3–7)	16.7%	No consensus
28. The medical oncologists must treat the pain of their cancer patients to the limit of their knowledge and capabilities before referring them to a pain unit	4 (2–7)	13.3%	No consensus
29. All cancer patients with pain should be seen at least once in a pain unit as part of comprehensive cancer care	3 (1–6)	53.3%	No consensus
30. Cancer patients may have their quality of life improved if they are sent early to pain units	7 (5–9)	63.3%	No consensus
**The intervention of the pain unit should be requested for a patient with cancer pain**			
31. When the pain is neuropathic or mixed	7 (5–8)	63.3%	No consensus
32. When the patient does not respond to the treatment prescribed by the oncologist	8 (7–9)	84.4%	Agreement in 1st round
33. As soon as the patient needs opioids	3.5 (2–5)	50.0%	No consensus
34. When there is poor control with high doses of opioids	9 (8–9)	87.5%	Agreement in 1st round
35. When there is significant toxicity with opioid treatment	8 (7–9)	81.3%	Agreement in 1st round
36. When an opioid rotation is required	5 (3–8)	26.7%	No consensus
37. When the patient is likely to benefit from an invasive technique	9 (9–9)	93.8%	Agreement in 1st round
38. Exclusively for invasive techniques	3 (2–7)	53.3%	No consensus
39. Only in the final stages of the disease	1 (1–2)	90.6%	Disagreement in 1st round
40. When functional improvement is not achieved	7 (6–9)	73.3%	Agreement in 2nd round
41. When there is an important psychosocial component	5 (3–7)	30.0%	No consensus
42. Who may benefit from infrequent or difficult to manage analgesic treatments	8,5 (7–9)	90.6%	Agreement in 1st round
43. With significant co-morbidities, where the choice of analgesic treatment and its follow-up is difficult	8 (7–9)	78.1%	Agreement in 1st round
**The recommended waiting time for oncology patients between an urgent request from the service of origin and their assessment by the pain unit should be**			
44. < 24 h	8 (5–9)	75.0%	Agreement in 1st round
45. < 48 h	8 (7–9)	87.5%	Agreement in 1st round
46. Pain management is never urgent	1 (1–1)	93.8%	Disagreement in 1st round
**The recommended waiting time for a cancer patient between the request for consultation from the service of origin on a preferential basis and its assessment by the pain unit should be**			
47. < 1 week	8 (7–9)	84.4%	Agreement in 1st round
48. < 2 weeks	9 (7–9)	76.7%	Agreement in 2nd round
49. < 4 weeks	1 (1–3)	78.1%	Disagreement in 1st round
**The recommended waiting time for cancer patients between the request for a routine consultation from the service of origin and their assessment by the pain unit should be**			
50. < 1 month	9 (6–9)	71.9%	Agreement in 1st round
51. < 3 months	4 (1–9)	6.7%	No consensus
52. < 4 months	1 (1–2)	84.4%	Disagreement in 1st round
**The pain follow-up of an oncology patient who is cared for in the pain unit should be carried out**			
53. By the doctor who sends the patient	5 (3–7)	33.3%	No consensus
54. By the pain unit	8 (6–9)	68.8%	Agreement in 1st round
55. By both in a consensual manner	9 (8–9)	96.9%	Agreement in 1st round
**In such cases that patients with cancer pain need a technique not available in their center**			
56. They should be referred directly from the pain unit to a unit where this technique is available	9 (8–9)	93.8%	Agreement in 1st round
57. They should be referred to their oncologist, who should organize the referral to the unit where the needed technique is performed, can be processed	2 (1–5)	66.7%	No consensus
58. It does not matter which specialty refers them	5 (2–9)	20.0%	No consensus

Referral criteria to a pain unit.

*IQR interquartile range.

**Table 3:** Block III results.

	Median (IQR)*	Degree of agreement	Results
**A frequent cause for not referring a patient to the pain unit when there is an indication for it, is**			
59. The delay until the first visit	7.5 (5–9)	60.0%	No consensus
60. Reassessments too far apart in time	7 (5–9)	60.0%	No consensus
61. The available pain unit offers few or no techniques	6.5 (3–8)	50.0%	No consensus
62. Lack of communication with the pain unit	8 (5–9)	68.8%	Agreement in 1st round
63. Refusal by the patients	2 (1–5)	71.9%	Disagreement in 1st round
64. The lack of pain unit availability in the hospital center	6.5 (3–8)	50.0%	No consensus
65. The previous bad experiences of the oncologist	5 (3–8)	36.7%	No consensus
66. The consideration that it does not confer any added value	5 (1–7)	30.0%	No consensus
67. Ignorance of the services portfolio of the pain unit	7.5 (6–9)	73.3%	Agreement in 2nd round
**In general, doctors working in a pain unit have sufficient training on**			
68. Cancer and its treatments, including its side effects	4.5 (2–6)	40.0%	No consensus
69. Communication techniques with cancer patients and their families	5 (3–6)	46.7%	No consensus
**In general, medical oncologists have sufficient training on**			
70. Pharmacological management of cancer pain	6.5 (5–8)	50.0%	No consensus
71. The use of non-pharmacological anesthetic techniques that may be available in a pain unit	4 (3–6)	43.3%	No consensus
**To improve the care of patients with cancer pain, the following is a priority**			
72. Improve coordination between medical oncology services and referral pain units	9 (8–9)	93.8%	Agreement in 1st round
73. Improve communication between medical oncology services and referral pain units	9 (8–9)	93,8%	Agreement in 1st round
74. Reduce the waiting time from the request for interconsultation until the patients are seen in the pain units	9 (8–9)	87.5%	Agreement in 1st round
75. Improve the infrastructure of the pain unit	9 (7–9)	78.1%	Agreement in 1st round
76. Expand the staff of the oncology services	6,5 (5–8)	50.0%	No consensus
77. Expand the staff of pain units	8 (7–9)	81.3%	Agreement in 1st round
78. Create interdisciplinary committees for cancer pain in hospitals that care for cancer patients	9 (7–9)	87.5%	Agreement in 1st round
79. It is necessary to establish systems for evaluating the quality of care for cancer pain agreed between the medical oncology services and the referral pain units	9 (8–9)	96.9%	Agreement in 1st round

Barriers and opportunities for cancer pain management improvement.

*IQR interquartile rang.

The questionnaire was submitted to the experts’ panel. Of the 35 panelists to which the items were sent, 30 responded to both evaluation rounds. Consensus was reached on 46 out of the 79 items evaluated in the first round. Additional 5 items reached consensus after the second evaluation round making a total of 51 out of the 79 proposed items (64.6%). Of them, 55 reached consensus on agreement and 6 on disagreement. The results of the items that reached maximal consensus are shown in Table 4.

**Table 4:** Results with maximal consensus.

There are many cancer pain patients who are not referred to pain units even though they could benefit from it	Agreement
**All hospitals that manage cancer patients must have**	
1. A pain unit of any kind, or failing that, a referral unit in another hospital center, where patients for pain treatment can be referred to	Agreement
2. A hospital oncological pain commission, where members of the oncology services and pain units can participate	Agreement
**The medical oncology service and the pain unit of a hospital center must have**	
1. Shared protocols for cancer pain management	Agreement
2. Consensual protocols for referring the patient to the pain unit	Agreement
In a hospital with a medical oncology and a pain unit, the doctors of the medical oncology service should be familiar with the portfolio of services of their referral pain unit	Agreement
Resident physicians of a medical oncology service should receive formal training in cancer pain pharmacological treatment	Agreement
The services portfolio of pain units should be public and easily accessible by any hospital specialty	Agreement
There are clear criteria for referring cancer pain patients to pain units	No consensus
**The intervention of the pain unit should be requested for a patient with cancer pain**	
1. When the patient does not respond to the treatment prescribed by the oncologist	Agreement
2. When there is poor control with high doses of opioids	Agreement
3. When the patient is likely to benefit from an invasive technique	Agreement
4. Only in the final stages of the disease	Disagreement
5. For patients who may benefit from infrequent or difficult to manage analgesic treatments	Agreement
The recommended waiting time for oncology patients between an urgent request from the service of origin and their assessment by the pain unit should be < 48 h	Agreement
Pain management is never urgent	Disagreement
The recommended waiting time for a cancer patient between the request for consultation from the service of origin on a preferential basis and its assessment by the pain unit should be < 1 week	Agreement
The pain follow-up of an oncology patient who is cared for in the pain unit should be carried out by both, the doctor who sends the patient and the pain unit in consensual manner	Agreement
In such cases that patients with cancer pain need a technique not available in their center they should be referred directly from the pain unit to a unit where this technique is available	Agreement
**A frequent cause for not referring a patient to the pain unit when there is an indication for it, is**	
1. Lack of communication with the pain unit	Agreement
2. Ignorance of the services portfolio of the pain unit	Agreement
In general, medical oncologists have sufficient training on the use of non-pharmacological anesthetic techniques that may be available in a pain unit	No consensus
**To improve the care of patients with cancer pain, the following is a priority**	
1. Improve coordination between medical oncology services and referral pain units	Agreement
2. Improve communication between medical oncology services and referral pain units	Agreement
3. Reduce the waiting time from the request for interconsultation until the patients are seen in the pain units	Agreement
4. Improve the infrastructure of the pain unit	Agreement
5. Expand the staff of pain units	Agreement
6. Create interdisciplinary committees for cancer pain in hospitals that care for cancer patients	Agreement
7. It is necessary to establish systems for evaluating the quality of care for cancer pain agreed between the medical oncology services and the referral pain units	Agreement

Table 5 summarizes the main statements agreed by the panelists and shows recommendations on the monitoring of the disease.

**Table 5:** Recommendations.

**All hospitals that manage cancer patients must have**
1. A pain commission with the participation of members of oncology and pain units
2. Either a pain unit or the possibility of referral to a pain unit in another hospital
**The medical oncology service and the pain unit of a hospital center must have**:
1. Shared protocols for cancer pain management
2. Shared clinical sessions to discuss cancer pain cases
3. Referral protocols to pain units
4. Cases should be discussed in the tumor committee
5. Oncologists should be familiar with the available services of the pain unit
6. Training should be organized for both oncologists and pain unit doctors
**Cancer patients should be referred to the pain units rather early or even before:**
1. The pain becomes uncontrollable,
2. High doses of opioids are required,
3. Complications or toxicity appear
4. Patients have significant co-morbidities
5. Patients may benefit from infrequent or difficult to manage analgesic treatments
**To improve the care of cancer pain patients**
1. Communication and coordination pathways together with interdisciplinary committees and care equality controls must be established between oncology and pain units
2. Consultation waiting times in pain units should be reduced
3. Staff and infrastructure of pain units ought to be expanded
4. Follow up should be done by both oncologists and pain unit doctors

Regarding cancer pain management (Table 1) the panelists agreed that there are many cancer patients not referred to pain units, and thus they do not receive relevant beneficial treatments. They recommended (Table 5) that in hospitals managing cancer patients at least a type II pain unit (unit with one or more healthcare providers with different professional training) must be available together with a pain commission and/or tumor committee where oncologists and pain specialists can discuss the management of cancer pain clinical cases. High quality communication and coordination regarding cancer pain management must exist between oncology services and pain units. In the case that such pain unit is not available, referral processes to a suitable unit in a different hospital must be in place.

Medical oncologist should be familiar with the services available in the referral pain units and both should organize training courses for their healthcare professional and trainees and issue regular updates regarding advances in their fields of practice aiming to keep healthcare professionals well informed and familiar with oncological and cancer pain treatments.

Regarding the referral criteria (Table 2), it appears that there are not clear-cut referral guidelines, as most of the assessed items did not reach consensus. The panelists agreed and advocated (Table 5) that cancer pain patients should be referred rather early to pain units and, most importantly, those with significant co-morbidities or difficult to manage analgesic treatments. Waiting times for urgent, preferential or routine referrals should be kept short. Moreover, referrals to pain units should be prioritized individually and they should be less than 48 h for urgent patients and less than 7 days for preferential patients. Oncologist and pain specialist should both follow up patients in a consensual manner by establishing appropriate circuits.

The panelists identified various barriers to an adequate management of cancer pain patients and also indicated improvement opportunities (Table 3). Lack of effective communication amongst healthcare professionals and with patients and their families, insufficient training, experience and skills in cancer and/or pain and their treatments and ignorance of the service portfolio of the pain unit were some of the deficiencies with a negative impact on the management of cancer pain patients.

In order to improve cancer pain care, they prioritized communication and coordination between oncology services and pain units, consultation waiting times reduction, infrastructure improvements and staff expansion of the pain units and interdisciplinary committees creation together with establishment of care quality evaluation systems for cancer pain (Table 5).

## Discussion

Approximately 10 million individuals are diagnosed with cancer yearly. Almost 70% of them will succumb to their disease or its complications and 60% will suffer from severe pain^14,15^. Epidemiological studies in Spain have also indicated that almost 55% of cancer patients experience pain^16^, frequently neuropathic in origin (20–33%)^17^ and 41% of cancer pain sufferers experience breakthrough pain^18^.

Since the introduction of the WHO´s analgesic ladder together with the American Pain Society recommendations on the assessment and quality indicators development for effective cancer pain management, an immense progress in oncological pain control has been achieved^19^. Remarkable efforts to improve pain management quality have been made; however, many cancer patients worldwide have their cancer-related pain inadequately managed^20^, causing poor life quality, anxiety and distress, depression and poor functional status^21^.

Traditionally, individual healthcare professionals, most often an oncologist, have managed cancer pain. However, cancer pain is complex and dynamic and therefore it requires a more thorough approach to assimilate the knowledge, experiences and skills of all those involved in its treatment via a multidisciplinary process with a shared philosophy, mission, and objectives^22^, preferably in specially created facilities, which allow for flexible collaborative pathways and movements among health care professionals. The International Association for the Study of Pain (IASP) proposed a four types (I-IV) classification of pain units depending on characteristics such as healthcare professionals participation, treatment options available, focus on pain types and education and research provisions^22,23^ The panelists agreed (Table 5) that all hospitals managing cancer patients must have at least one type II pain unit, a service of several unidisciplinary specialists for the treatment of pain and highlighted the importance of being able to refer patient from a unit with less facilities to a better one, where pain can be managed more adequately.

The importance of a multidisciplinary approach to the care of cancer pain has been emphasized by a recent Spanish quality recommendations publication on oncological pain management (Norma ACDON), which besides a shared philosophy, mission and objectives, is characterized by several other aspects: interdependence among team members, mutual respect, open communications, cooperation and diverse viewpoints being some of the most important ones^24^. It is necessary that all teams and disciplines involved collaborate and coordinate their activities and combine their skills and experience effectively towards a common goal. Importantly, a delivery system must be in place, which promotes and facilitates execution^22^.

Both patient-centered care and continuity of care are fundamental for coherent and consistent interventions directed to cancer patients´ medical and personal needs^25^. Constant communication, not only among the team members but also with the patients and their families by encouraging active participation, is a central element for coordinated care and clinical and mental status improvement of the patient by focusing on specific issues furthering common aims and managing pain and possible side effects in a holistic and multidisciplinary way.

Critical for effective communication and collaboration among all those involved in this cancer pain multidisciplinary care is accurate and detailed care documentation, through which, progression towards shared short and long-term management targets can be established. This, together with a proper follow-up schedule is a useful tool to substantiate effectiveness of treatment, detect therapeutic issues and prevent and manage complications and relapses^22^. Shared clinical sessions for cancer pain cases discussion, shared cancer pain management protocols, common referral pathways arrangements and extensive and constant contact possibilities via telecommunication facilities for case consultation are essential requirements for a high quality care of cancer pain patients and they have been strongly recommended by the panelists (Table 5).

The panelists also identified those patients that may benefit the most by this multidisciplinary approach when treating oncological pain: patients with high doses of opioids, or with hyperalgesia, those that require or may benefit from interventional techniques, those with co-morbidities or important side effects from analgesic treatments and with advanced disease and major pain.

Another area of attention highlighted by our experts was training and education of oncologist and pain healthcare professionals and research (Table 5). Lack of knowledge regarding cancer treatments and cancer pain management amongst the members of the multidisciplinary team has been reported as a cancer pain poor management factor in clinical practice^8^. Interestingly, often healthcare professionals do not recognize their cancer management knowledge deficits^4^. There is a need for continuing education and training in: pain management techniques (both, pharmacological and interventional), psychological support, pain topics such as controlled analgesia methods, pain evaluation scales, questionnaires for practicing physicians, trainees of medical oncology units and similarly cancer therapeutic modalities and communication skills with cancer patients and their families for the pain services providers, multidisciplinary cancer and cancer pain rounds where cases can be discussed and most importantly familiarity of the involved parties with portfolios of the available services.

Deciding who, how and when to refer cancer pain patients to pain units can be quite complex and factors such as resources availability, involved teams capacities, disease characteristics, levels of care provided and health care policies may play an important role^26^. The complexity of this process is also displayed in our panelists’ answers, where 41% of the items regarding referral criteria to pain units did not reach consensus.

Referrals to pain units can be either oncologist-driven or automatic. Oncologist-driven referrals require identification of patients with specific symptomatology and care necessities, and are subject to varying thresholds the referring physicians might have depending on their knowledge, experience and skills^27^. Often this referral pattern leads to treatment delays.

In the automatic referral process pre-established criteria act as consultations triggers with pain units depending on patients clinical needs. It appears that this referral pattern has the potential to streamline cancer pain management and, in general, palliative care^28^. The items that reached consensus could be used as the triggering criteria to structure this referral pattern.

As it has been mentioned already, 33% of cancer pain patients are not controlled effectively^2^. Recent studies have identified various barriers to their adequate treatment, such as insufficient understanding and experience with the available therapeutic options and approaches to the evaluation and management of cancer pain, lack of coordination and cooperation amongst the various disciplines involved in the management of these patients, often inadequate pain management resources in cancer units, inefficient communication flows and referral patterns between oncology and pain control units, anxiety for opioid use side effects and patients misconceptions regarding analgesic usage^29^.

Furthermore, barriers in cancer pain management may be related to healthcare professionals, patients themselves or the existing healthcare system (limited access to specialist’s services). The most frequently mentioned barrier related to the clinicians is inadequate pain evaluation. Pain assessment can be very subjective and only a small percentage of physicians use the available pain assessment tools (like the visual analog scale VAS) routinely for an accurate evaluation^30^. Clinicians are often reluctant to start opioid treatments and tend to use them only in terminal cancer or intractable pain cases^10^. Finally, lack of specific knowledge concerning medications for chronic cancer pain, pain pathogenesis, dose titration, breakthrough pain, addiction and tolerance appears to be another barrier in the effective treatment of these patients^31^.

Patients´ perspective barriers can be of cognitive or affective nature or related to non-adherence to analgesics. Under cognitive barriers we see situations where painkillers concern and misconceptions lead to pain underreporting, inadequate communication with clinicians and side effects fear, addiction and tolerance^32^. Depression, stress and anxiety are affective barriers that alter pain perception and predict treatment responses^33^. Finally, adherence to pain medication is positively related with better pain control^34^.

A series of interventions have been proposed to overcome all these barriers between oncology and pain management units with the aim to facilitate patient flow, minimize waiting times and achieve adequate cancer pain control: effective pain assessment by using appropriate validating tools and multidimensional evaluations; pain management according to the published guidelines, by using the indicated medications, monitoring outcomes and side effects in a multidisciplinary approach; educating health care professionals and trainees through continuing medical education, lectures and interdisciplinary pain management rounds; educating patients and their families by informing them regarding their disease and appropriate medication use and providing psychosocial support and finally, healthcare system-based issues can be better controlled by increasing pain and palliative services^4^. Our experts have also highlighted the importance of the above interventions during their assessment in order to improve the management of cancer pain and consequently the patients’ wellbeing (Table 5).

There are some limitations of our work that ought to be mentioned. The consensus methodology prevents in depth discussions and some matters may be overlooked. In addition, subjectivity linked to personal evaluations may be a problem, and there is a potential bias in the selection of the expert panel. However, panelists were selected taking into account their contrasted clinical experience and expertise in cancer field, and in our opinion their point of view could be helpful for the rest of the health providers. Further studies may be carried out to identify impediments and approaches to overcome these barriers from the point of view of other healthcare professionals such as nurses specialized in cancer or primary care physicians.

## Conclusions

In summary, the results of this survey identified patterns of effective collaboration between oncology and pain units, referral criteria regulating cancer pain flow among the involved services in a multidisciplinary way, and the main barriers to optimal cancer pain management. Also practical recommendations to overcome these barriers were proposed. This consensus may be useful for clinicians and health managers to implement measures aimed to improve cancer patients’ pain management.

## Data Availability

All data generated or analysed during this study are included in this published article.

## References

[1] Sayed D (2013). The interdisciplinary management of cancer pain. Tech. Reg. Anesth..

[2] Breivik H, Cherny N, Collett B (2009). Cancer related pain: A pan-European survey of prevalence, treatment, and patient attitudes. Ann. Oncol..

[3] Van den Beuken-van EM, De Rijke J, Kessels A (2007). Prevalence of pain in patients with cancer: A systematic review of the past 40 years. Ann. Oncol..

[4] Kwon J (2014). Overcoming barriers in cancer pain management. Clin. Oncol..

[5] Perez J, Olivier S, Rampakakis E, Borod M, Shir Y (2016). The McGill university health centre cancer pain clinic: A retrospective analysis of an interdisciplinary approach to cancer pain management. Pain Res. Manag..

[6] Smith T, Staats P, Deer T (2002). Randomized clinical trial of an implantable drug delivery system compared with comprehensive medical management for refractory cancer pain: Impact on pain, drug-related toxicity, and survival. J. Clin. Oncol..

[7] Hong S, Roh S, Kim S (2010). Change in cancer pain management in Korea between 2001 and 2006: Results of two nationwide surveys. J. Pain Symp. Manage.

[8] Deandrea S, Montanari M, Moja L (2008). Prevalence of undertreatment in cancer pain: A review of published literature. Ann. Oncol..

[9] González-Escalada J, Camba A, Casas A, Gascón P, Herruzo I, Núñez-Olarte J, Ramos-Aguerri A, Trelis J, Torres L (2011). Good practice code for the control of oncological pain. Med. Paliat..

[10] Breuer B, Fleishman S, Cruciani R (2011). Medical oncologists’ attitudes and practice in cancer pain management: A national survey. J. Clin. Oncol..

[11] Fennell M, Prabhu Das I, Clauser S, Petrelli N, Salner A (2010). The organization of multidisciplinary care teams: Modeling internal and external influences on cancer care quality. J. Natl. Cancer Inst. Monogr..

[12] Dekel B, Varani S, Robert SamolskyDekel A, Di Nino G (2014). Melotti pain control in the continuity of care. R CMI.

[13] Diamond IR, Grant RC, Feldman BM, Pencharz PB, Ling SC, Moore AM (2014). Defining consensus: A systematic review recommends methodologic criteria for reporting of Delphi studies. J. Clin. Epidemiol..

[14] Callaway M, Ferris FD (2007). Advancing palliative care: The public health perspective. J. Pain Symptom Manage.

[15] Stjernsward J, Foley KM, Ferris FD (2007). The public health strategy for palliative care. J. Pain Symptom Manage.

[16] Porta-Sales J, Nabal-Vicuna M, Vallano A, Espinosa J, Planas-Domingo J, Verger-Fransoy E (2015). Have we improved pain control in cancer patients: A multicenter study of ambulatory and hospitalized cancer patients. J. Palliat. Med..

[17] Perez C, Sanchez-Martınez N, Ballesteros A, Blanco T, Collazo A, Gonzalez F (2015). Prevalence of pain and relative diagnostic performance of screening tools for neuropathic pain in cancer patients: A cross sectorial study. Eur. J. Pain..

[18] Pérez-Hernández C, Blasco A, Gándara Á, Mañas A, Rodríguez-López MJ, Martínez V, Fernandez-Nistal A, Montoto C (2019). Prevalence and characterization of breakthrough pain in patients with cancer in Spain: The CARPE-DIO study. Sci. Rep..

[19] Gordon D, Dahl J, Miaskowski C (2005). American Pain Society recommendations for improving the quality of acute and cancer pain management: American Pain Society task force of care. Arch. Intern. Med..

[20] Butt S, Tarar W, Amin F (2013). Pain management in cancer patients in tertiary care hospitals. JPMI.

[21] Green C, Hart-Johnson T, Loeffler D (2011). Cancer-related chronic pain: examining quality of life in diverse cancer survivors. Cancer.

[22] Turk D, Stanos S, Palermo T (2010). Interdisciplinary Pain Management.

[23] Palanca Sánchez, I., Puig Riera de Conias, M., Elola Somoza, J., Bernal Sobrino, J., Paniagua Caparrós, J. Grupo de Expertos. Unidad de tratamiento de dolor: estándares y recommendaciones. Ministerio de Sanidad, Política Social e Igualdad, 2011. Recuperado. http://publicacionesoficiales.boe.es/detail.php?id=055986011-0001. Accessed 28 Aug 2018.

[24] Pérez C, Martin-Delgado J, Vinuesa M, Ibor PJ, Guilabert M, Gomez J, Beato C, Sánchez-Jiménez J, Velázquez I, Calvo-Espinos C, Cánovas ML, Yáñez JA, Rodríguez M, Baquero JL, Gallach E, Folch E, Tuca A, Santiña M, Mira JJ (2021). Pain standards for accredited healthcare organizations (ACDON project): A mixed methods study. J. Pers. Med..

[25] Haggerty J, Reid R, Freeman G (2003). Continuity of care: a multidisciplinary review. BMJ.

[26] Hui D, Bruera E (2016). Integrating palliative care into the trajectory of cancer care. Clin. Oncol..

[27] Schenker Y, Crowley-Matoka M, Dohan D (2014). Oncologist factors that influence referrals to subspecialty palliative care clinics. J. Oncol. Pract..

[28] Bakitas M, Tosteson T, Li Z (2015). Early versus delayed initiation of concurrent palliative oncology care: Patient outcomes in the ENABLE III randomized controlled trial. J. Clin. Oncol..

[29] Pargeon K, Hailey B (1999). Barriers to effective cancer pain management: a review of the literature. J. Pain Symptom Manag..

[30] Silvoniemi M, Vasankari T, Vahlberg T (2012). Physicians’ self-assessment of cancer pain treatment skills: More training required. Support Care Cancer.

[31] Gallagher R, Hawley P, Yeomans W (2004). A survey of cancer pain management knowledge and attitudes of British Columbian physicians. Pain Res. Manag..

[32] Ward S, Goldberg N, Miller-McCauley V (1993). Patient-related barriers to management of cancer pain. Pain.

[33] Wang H, Kroenke K, Wu J (2012). Predictors of cancer-related pain improvement over time. Psychosom. Med..

[34] Jerant A, Franks P, Tancredi DJ (2011). Tendency to adhere to provider-recommended treatments and subsequent pain severity among individuals with cancer. Patient Prefer Adherence.

